# Insect Food Products in the Western World: Assessing the Potential of a New ‘Green’ Market

**DOI:** 10.1093/aesa/saz015

**Published:** 2019-09-11

**Authors:** C Matilda Collins, Pauline Vaskou, Yiannis Kountouris

**Affiliations:** Centre for Environmental Policy, Imperial College London, The Weeks Building, London, United Kingdom

**Keywords:** entomophagy, marketing, acceptability, sustainable food, willingness to pay

## Abstract

Although two billion people already eat insects in the world and the benefits of edible insects are well known, these ‘green’ sources of protein are neither treated as conventional food products nor widely incorporated into Western diets. Using a school-based investigation surveying 161 children, aged 6–15, and 114 of their parents in London, and an online consumer survey with mainly British and French consumers (*N* = 1,020), this research provides insights into the potential of the insect market in the West. This work supports the idea that incorporating insect food into our diets makes not only environmental but also business sense. A nonnegligible segment of the population surveyed is willing to pay for mealworm minced meat and young children and pre-teens could represent a substantial market segment, as yet unexplored. This analysis points to multiple marketing strategies, such as early exposure, education, reducing the visibility of insect parts, celebrity endorsement, or peer-to-peer marketing, all of which could facilitate the adoption of insect food in the ‘mainstream’ arena, according to the consumer segment being targeted. Generalizations from these results are restricted to an educated and youthful subset of the potential consumer pool and further work remains to understand the patterns of Western consumer acceptance for the range of insect foods.

Entomophagy, the practice of eating insects, is traditional in many cultures but not in Western countries (Yen 2009). It is common practice for at least two billion people and there are now more than 2,000 recorded species of edible insects around the globe (Ramos-Elorduy 2009, van Huis 2013). Edible insects, such as crickets and grasshoppers (orthopterans), mealworms (coleopteran larvae), caterpillars (lepidopteran larvae), or various fly larvae (dipterans), could provide an alternative meat source, while improving our food security. They have been put forward as a sustainable way to support growing meat consumption, food security, as well as promoting healthier diets. Edible insects are, in general, rich in protein, fat, and energy and can also be a significant source of vitamins and minerals (Rumpold and Schlüter 2015).

By 2050, world meat consumption is predicted to increase by about 44% compared with 2005 figures (Alexandratos and Bruinsma 2012). Current food production systems may not sustain both the projected world population and the projected consumption patterns of meat products, in particular in the context of climate change (Brown and Funk 2008). Excluding insects from our diets has been said to be irrational in the context of food shortages, price spikes, and may put the resilience of food supplies at risk (Premalatha et al. 2011). Some insect species could be used in Western countries as alternative protein sources, in particular with the aim to ensure the security of the meat-protein supply (van Huis 2013). The contribution that insects make to food security in Africa, Asia, and South America has been documented, but in the 20th century, little attention was paid to the role they could play as protein contributors in the Western world (Illgner and Nel 2000).

High intakes of red meat and poultry are associated with increasing economic development but have costly environmental impacts in terms of greenhouse gas emissions (GHG), water use, and land area needed (Smil 2002, Dibb and Fitzpatrick 2014). Switching some of our traditional sources of protein to insect-based protein is one way to make our food production more sustainable. Insects can be raised in smaller spaces with lower water and energy inputs per g/protein than those required for other animal species. They also produce fewer greenhouse gases and ammonia, limiting soil nitrification and acidification (Oonincx et al. 2010). Efficiency is intrinsic to raising insects as they can convert feed 12 times more efficiently than cattle (van Huis et al., 2013) and 80–100% of their body mass is commonly used compared with 40% for beef (Nakagaki and Defoliart 1991, van Huis 2013). Insects are the most speciose group on earth (May 1988) and they grow much faster than poultry, beef, veal, sheep, or pig meat. For example, the drugstore beetle can provide protein for 100 people in only 40 m^2^ (Kok 1983). Insects, thus, have the potential to provide sustainably sourced food in quantity as well as in a variety of flavors and textures.

Insects are considered nutritionally healthier than red meat (Bukkens 2005, Magalhães et al. 2012) and including them in our diets may decrease risk factors for some diseases as well as generate an environmental quality gain. Dietary changes that reduce GHG emissions can also generate additional health co-benefits (Milner et al. 2015). Besides providing a high-protein food source, insect food may also be useful in tackling the most common micronutrient deficiencies in developed countries: iron, vitamin A, and zinc; thus, making insects part of our dietary patterns could be expected to result in substantial public health savings (Müller and Krawinkel 2005, Bates et al. 2012, Finke 2013).

The Western attitude to insect eating has additional far reaching social effects which can result in reduced use of insects in regions where their nutritional input is crucial (DeFoliart 1999). As a result, normalizing insects in the West can be part of a wider effort to preserve traditional diets and limit the worldwide obesity and malnourishment epidemic (Popkin 2003).

## The ‘Disgust Factor’

The modern human already eats insects invisibly: the U.S. Food and Drug Administration reports that there may be 60 fragments of insect in 100 g of chocolate for example, and the idea of eating insects is far from new, but the very slow uptake in the West suggests that there are considerable barriers (Holt 1885, Shelomi 2016, U.S. FDA 2018). Cultural disgust is thought to be the main reason that insects are not part of the diet in Europe and North America and strong cultural barriers to their widespread adoption in the West have long been documented (Vane-Wright 1991). Indeed, the Chinese consistently give higher ratings to insect food compared with Germans and insects are a delicacy in many parts of the world; grasshoppers cost more than goat meat in Uganda, for example (Agea et al. 2008, Hartmann et al. 2015). Nutritional arguments are not thought to be enough to overcome the ‘disgust factor’ and convert Westerners to insect-based dishes (Deroy et al. 2015).

## A Rising Interest

Although eating insects is still rare in Europe and North America, there has been a rising interest since the turn of the millennium (Tucker 2013). The Food and Agriculture Organization of the United Nations catalyzed this interest in a report advocating the use of insects in food and feed, and the European Union (EU) supported the first international conference on insects to feed the world in 2014 (van Huis et al. 2013, van Huis 2015). The field is maturing, and there are now formal bodies, such as the International Platform of Insects for Food and Feed (IPIFF) in Europe and the North American Coalition for Insect Agriculture (NACIA) cohering and supporting the production, regulation, and marketing of insect-derived protein. Commercially, mealworm burgers have been sold in Holland since October 2014, the U.S. fast food chain Wayback Burgers first included cricket milkshakes on its 2015 summer menu, and the European supermarkets Carefour and Sainsburys are now stocking insect snacks. Consequently, academic, political, and market dynamics indicate a growth opportunity for insect food in the future.

## Novel Food Experiences

Several factors influence openness to new food experiences, for example, expectations of food flavor (Yeomans et al. 2008) and/or fear of the new food (Pliner and Hobden 1992). Cultural background and individual experience are also particularly relevant in the case of insect food. Thai participants were less reluctant to try dishes with visible insects compared with Dutch participants in a focus group (Tan et al. 2015). Influential factors may still be changed, and with appropriate marketing, Westerners could reverse their acquired distaste (Hobden and Pliner 1995, Deroy et al. 2015).

Factors that may help adoption of new foods are as follows: information about the benefits of the product (Fischer and Frewer 2009), repeated exposure to the product (Birch and Marlin 1982), familiar flavors (Caparros Megido et al. 2014), ‘naturalness’ of the product, and ‘trust’ (Siegrist 2008). Insect food processed to resemble familiar items may be more palatable to many relative to unprocessed insects (Hartmann et al. 2015). The past tells us that protein initially seen as unconventional may become popular; sushi in the West (Bestor 2000), and the American experience of lobster, for example (Luzer 2013). There is increasing evidence that Westerners’ food tastes are changing rapidly; they are expanding the range of food seen as edible, which could potentially include insects (Tucker 2013).

The potential for insects to become ‘mainstream’ in Western markets is hard to generalize from the available studies which show mixed results. Insect-eating was qualified as ‘promising’ (Caparros Megido et al. 2014), but this study sampled only participants with a previous interest in insects. Opportunities for introducing insects to Belgian consumers were deemed nonexistent by Vanhonacker et al. (2013), though consumer acceptance may have improved since, thanks to extensive media coverage and a public increase in environmental awareness. One-third of a representative U.S. population sample recently declared being likely to buy insect-based products (TrendsTracker (Blueshift Research) 2015) and one out of five meat consumers have said that they are ready to adopt insect food (Verbeke 2015). Despite this, it is considered that the diffusion of entomophagy has so far ‘failed’ (Shelomi 2016). Growth in the insect market, thus, asks for a different perspective including different consumers and building on market research.

## Consumer and Market Research

Most Western consumer surveys have been limited to a small number of Dutch, Belgian, and Australian consumers (Lensvelt and Steenbekkers 2014). Nevertheless, it seems that younger males may be more likely to adopt insect food (Verbeke 2015), and this suggests that targeting specific population segments may be successful. Similarly, it was found that respondents aged 18–29 yr were the age group most likely to buy insects (TrendsTracker (Blueshift Research) 2015). Additionally, anecdotal evidence hints that targeting children may be a good way to encourage a new generation to eat insects (Vane-Wright 1991, Nacson-Jones 2014), as their food preferences may be more malleable, but the published studies on insect food have included few individuals under the age of 20 yr.

Marketing strategy would benefit from more research on how Western taste can be influenced. Insect food presented to survey participants has usually been restricted to insect-based products viewed in isolation, whereas Elzerman et al. found that meal combinations were important in rating meat substitutes (2011). Educational ‘bug banquets’ may seem like an easy way to introduce people to entomophagy, but their effect on attitudes has not been straightforward (Looy and Wood 2006). Previous literature gives us reason to expect that the visibility of insects has a shaping influence on Westerners’ willingness to try. Reducing insect visibility when there was no previous experience of the species increased food appreciation (Tan et al. 2015), but Lensvelt and Steenbekkers also found that some consumers were specifically interested in eating whole animals (2014). This area remains full of mixed messages and recent work examining the idea that utilitarian claims (health and environment) would promote consumption has found that a focus on these may, in fact, decrease consumption in comparison to immediate, hedonic claims (e.g., tasty; Berger et al. 2018).

A principal concern for many consumers is food prices (Hayes et al. 2014) and as meat prices rose by 35% between 2007 and 2014, it is likely that price can play a key role in the growth of the market, but no study has yet looked at insect protein in relation to Willingness to pay (WTP) or price/visibility trade-offs.

## Methods

Using a mixed-method approach, we sought to provide insights into the potential of insect food to grow market share in Europe. We have 1) evaluated whether urban children are more likely to start eating insects than adults, 2) surveyed potential Western consumers with an online consumer survey to begin to understand patterns of WTP and the trade-offs made when considering insect food products, and 3) assessed several different marketing strategies for edible insects (snack format, promotion with insect bars, celebrity endorsement, etc.).

### School Investigation

Two London schools participated in this project (Table 1). The ethical aspects of the school investigations were considered and discussed with the school representatives before the project was approved by the Centre for Environmental Policy (CEP). Eight school classes took part in one or two sessions, as some requested a follow-up visit to discuss research and scientific method as part of an extended outreach program. Questionnaires were reviewed by the school staff and parents were contacted prior to the activity and voluntary consent for their children’s participation was obtained as per guidelines.

**Table 1.** Descriptors of the two schools visited. Income was estimated from the mean household income of the five surrounding wards for each school

Characteristics	School A	School B
Years of schooling	primary school	secondary school
Age of children visited	6–12 yr	13–16 yr
Typical class size	17–20 pupils	28–32 pupils
Education type	Partly girls-only classes	Fully mixed-gender
Mean local household income	£62,000 per year	£38,000 per year

#### In-class Activity

Before the topic was introduced, pupils were asked if they knew that insects could be eaten for food. They were also asked, while their eyes were closed to reduce any peer pressure, to raise their hands if they would eat insects both before and after the session. The main benefits of insect food were then briefly introduced, following a general script used with all classes, before setting up a group activity. Groups of three to five pupils, seated together at small tables, agreed the distribution of 20 paper clips between four images of insect food. The more paper clips an image got, the more appealing it was judged to be. The sets of four images each group received were created by randomly selecting one of three possible from each of the four categories of insect food: ‘snacks with visible insects’, ‘snacks with no visible insect’, ‘dishes with visible insects’, and ‘dishes with no visible insect’ (see Supp Materials [online only]). Each group was finally asked to write a sentence under each picture summarizing the reason for their choice.

#### School questionnaires

Pupils then completed a one-page questionnaire exploring their opinion of insect food and each child took another similar questionnaire home for their parents to complete (see Supplementary Materials). The parents’ questionnaires included a text description of the benefits of insect food and insect food images, so that parents could answer after having been given comparable information to their children. The questionnaire design was checked using the checklists proposed by Denscombe for small-scale social research projects (2014) and recommendations from Peterson (2000). Answers to opinion questions were given using a negative-to-positive scale, often used in Q methodology (McKeown and Thomas 2013). Respondents scored items from −5 to +5, with no opinion or unsure rated ‘0’. Balanced written anchor points were given so that each respondent would see the scale in the same way. Answers to the schools’ questionnaires and the online survey were entered on Microsoft Excel. Analysis used the R software environment (R Core Team 2015).

### Online Consumer Survey

#### Format and Design

An online consumer survey designed with Qualtrics software (Smith et al. 2015) was distributed from 17 June to 12 July 2015. Although 14% of households in Great Britain do not have internet access and daily computer use is lower for people over 65 yr compared with the rest of the population (ONS,UK 2015), a web format was used to increase sample size and coverage. The wording, layout, and general design of the survey were based on tailored survey methodology adapted for internet surveys (Dillman et al. 2014) to improve the quantity and quality of response. It was also informed by the preliminary results from the initial responses in the school activity and the school questionnaires and was written in English and French.

Distribution was by convenience sampling (Imperial College mailing lists, social media, personal contacts, etc.) and a ‘snowball effect’ was used to increase the sample size by asking respondents to share the survey link. A prize draw of £25, or the euro equivalent, was selected as an incentive to take part, based on social exchange theory (Singer and Ye 2013). To reduce bias, the survey was introduced by a message asking potential respondents to help knowledge on ‘the future of food’. People already interested in insect food may have been more likely to respond otherwise (Groves et al. 2004). The order of possible answers for opinion questions was randomized, as well as the order of questions themselves when logically possible, to eliminate position effect (Krosnick and Alwin 1987). To mitigate response bias, participants were reminded that the survey was anonymous, that it was part of a student-led research project and they were also asked to be honest in their answers.

#### Contingent valuation scenario

To elicit potential consumers’ WTP for insect protein, a Contingent Valuation approach was implemented. The valuation scenario described the projected under-supply of meat; health and environmental benefits of insect protein were outlined and examples of edible insect species were given (see Supp Materials [online only]). WTP was elicited using a payment scale with prices ranging from £1 to £9 in £0.10 increments. WTP was converted to a continuous variable using interval midpoints. Before responding to the WTP question, respondents were reminded to consider their income and expenditure on similar goods and services. Debriefing questions allowed the identification of protest responses which were then excluded from the estimations, as suitable for a private good model analysis (Lindsey 1994). The econometric analysis used Stata 11 software (StataCorp 2009) and a 0.729 conversion rate between euros and pound sterling. To assess the influence of sociodemographic characteristics on consumers’ WTP, accounting for the self-selection in the market participation decision, a Heckman two-step model (Heckman 1979) was estimated.

The regression model accounted for standard sociodemographic characteristics known to affect consumer purchasing decisions including gender, age, income, and nationality. Psychographic variables, such as the frequency of green shopping, the frequency of exercise, and meat-eating behavior, were also controlled for. People who exercise more may be more likely to care about the protein profile of their food and so may be expected to be more positive toward insect food. Greener shoppers may also be more willing to pay for environmentally friendly insect protein sources. On the other hand, vegetarians may be less willing to try insect food compared with regular meat-eaters. Previous experience with insect protein was also expected to have a positive effect on WTP.

#### Choice Experiment

A choice experiment was implemented to assess the relative importance and estimate consumers’ WTP for attributes charactering an insect protein product. Specifically, the insect protein product was characterized by the following attributes: 1) insect visibility, 2) readiness to eat, and 3) price. The first two attributes were binary in levels. Insects could be visible or not and protein could be ready to eat or not (e.g., required cooking). The price attribute had seven levels: £5.00, £4.90, £4.80, £3.50, £3.20, £1.40, and £1.00. Respondents were presented with four choice sets, each containing three alternatives composed of the attributes at different levels: two opt-in alternatives and one opt-out ‘not buy’ alternative (see Supp Materials [online only]). To facilitate consumers’ choice, opt-in alternatives were presented through images of insect meals. Presenting food in a meaningful way to the participants helped give an accurate picture of its acceptability (Marshall and Bell 2003). Data were analyzed with a conditional logit model (McFadden 1972).

## Results

### School Investigation

#### Demographics and Background Questions

One hundred and sixty one children, 65% boys and 35% girls, took part in the school-based activity and associated survey. One hundred and fourteen parents, 58% women and 42% men, of these children returned a survey. The age of adult respondents ranged from 33 to 75 yr with a mean of 45 yr (SD = 6.27). School A, associated with a higher-income neighborhood, had a household response rate of 72% compared with 11% for the other. More than 68% of children and 95% of adults knew that insects can be consumed as food. About 16% of children and 29% of adults had eaten insects before and <2% did not eat meat.

#### Insect Food Pictures

**Fig. 1.**
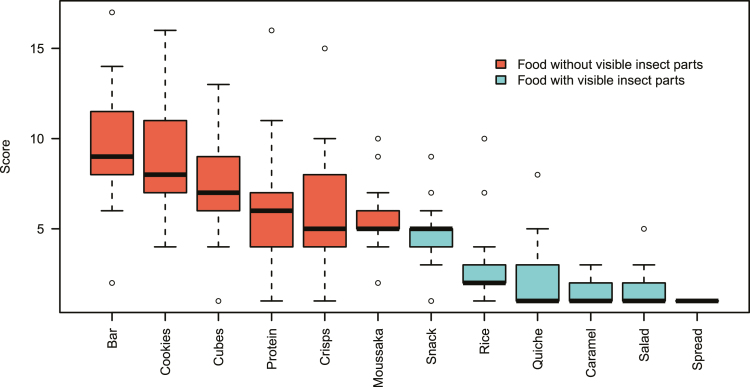
Ranking of insect food pictures (red/darker = food without visible insects, blue/lighter = food with visible insects or insect parts in order of preference: insect bar, cookies with cricket flour, insect cubes, mealworm protein with rice, cricket crisps with some of the ingredients, grasshopper moussaka, insect snacks, fried rice with larvae, insect quiche, caramel-dipped locusts, bug salad, insect spread, see Supp Information [online only] for the pictures).

Pictures of food without visible insects or insect parts were preferred over pictures with visible insects (t^136^ = 12, *P* < 0.001, Fig. 1). No preference was identified between pictures of snacks and meals (t^165^ = 1.6, *P* > 0.05), nor was a stated preference for one over the other seen in the questionnaire (χ^2^ = 1.2, df = 1, *P* > 0.05). Groups of children wrote 216 explanatory notes in total, one for each insect food picture they were given; 179 readable and informative comments were available for thematic analysis (see Table 2).

**Table 2.** Recurring themes from the children’s comments on insect food pictures

Theme
‘**Ranking the food in relation to other food**
‘They are cookies and I love cookies’.
‘I would eat it if it was dipped in chocolate’.
**Ability to distinguish insect parts**
‘I like this cookie with cricket powder because it doesn’t show any bug’.
‘You can identify the insects it contains, off putting!’
**Presentation of the food**
‘It looks appetising and is presented professionally’.
‘It doesn’t look appealing, it would be better if it was put in snack form’.
**Anticipated texture of the food**
‘It looks quite crispy, I think I would like to have a try’.
‘It looks slimy and not tasty’.
**Looking like usual food**
‘It looks very appetising as it looks like a normal cereal bar’.
‘We would eat this because it looks like our regular food’.
**Expected taste of the food**
‘It is powder so you wouldn’t taste it as much’.
‘They look very nice as they have a BBQ flavour’.
**Looking ‘alive’ or ‘moving’**
‘It has insects crawling all over it’.
‘You can see the bug and its eyes’.
**Nutrition-related comments**
‘It looks like a nice healthy, nutritious meal’.
‘It has healthy things on it’.

#### Children and Parent Surveys

No variation was detected between children and parents in the stated willingness to try or have insect food regularly. However, willingness to try insect food decreased with age in children (rs^159^=−0.26, *P* < 0.01), unlike for adults where no variation was identified with age. As a result, younger children, up to 11-yr old, were found to be more likely to want to try insect food relative to adults (t^136^ = 2.7, *P* < 0.01). They were also positively influenced by their peers when discussing insect food pictures (t^63^ = 4.8, *P* < 0.001), whereas older age groups reported a negative effect of peer conversation (t^96^ = −3.2, *P* < 0.01).

Overall, these respondents were ‘unsure’ about their openness to trying insect food; both the scores of children (t^160^ = −1.56, *P* > 0.05) and parents (t^113^ = −2.0, *P* > 0.05) were not different from ‘0’. Children (t^160^ = −6.8, *P* < 0.001) and parents (t^113^ = −7.7, *P* < 0.001) both gave negative answers when asked if they could imagine regularly eating insects. Indeed, children (t^160^ = 3.5, *P* < 0.001) and parents (t^113^ = 3.6, *P* < 0.001) were both less likely to imagine regularly eating insects compared with simply trying insect food.

A greater proportion of children surveyed, 56%, would eat insect food after the information session than before (χ^2^ = 35.2, df = 1, *N* = 161, *P* < 0.001). Parents who had tried insects before (t^37^ = 4.1, *P* < 0.001), and even more so children (t^119^ = 2.0, *P* < 0.05), were more likely to want to try insect food over others with no previous experience. Women had more negative attitude than men (t^119^ = −3.6, *P* < 0.001), who felt, on average, neutral about insect food (t^59^ = 1.3, *P* > 0.05). Conversely, though girls were unsure about trying insect food, boys were slightly resistant (t^114^ = 2.2, *P* < 0.05).

Parents who answered positively to caring about whether the food they buy is ‘green’ were not seen to be more likely to want to eat insects and although parents leaned to believing that insect food could help feed the world, with an average of 1.80 on the ±5 scale (SD = 2.42), no correlation was seen between willingness to try insects and this. Willingness to try was not seen to vary between British and non-British participants and no correlation was seen between enthusiasm for insect food and a stated enjoyment in trying new things (*P* > 0.05 in all cases).

### Online Consumer Survey

#### Sample Description and Background Questions

In total, 1,020 people took part in the online survey from 17 June to 12 July 2015, with a 92% completion rate. About 54% of respondents answered the French version, whereas the rest answered the survey in English. About 95% of respondents indicated living in Europe, the United States or Canada with half from France, nearly a third from the United Kingdom and 7% from the United States.

About 65% of the respondents were female and 85% had an undergraduate degree. The median age was 21 yr with 45% of respondents over 25-yr old and an age range from 12- to 90-yr old. The mid-point income bracket mean indicated a typical annual income of £40,000.

The vast majority of respondents were meat-eaters; environmental arguments were given by 40% of the non-meat-eaters. About half of the sample indicated exercising more than once a week and one in four respondents indicated buying organic or eco-friendly products a few times a month. About 97% of participants were aware that insects can be cooked for food. More than one-third had eaten insects before and had, on average, enjoyed the experience (mean = 1.37, SD = 2.31).

#### Contingent Valuation: Willingness to Pay

The coefficient estimates of the WTP linear regression can be found in Table 3 and the final model estimated with the two-step Heckman method is summarized in Table 4. The variable ‘high frequency of green shopping’ was generated combining answers from people buying organic or eco-friendly food products a few times a month, once a week, and every time they shop. The variable ‘high education level’ is binary, equal to 1 for respondents that completed tertiary education.

**Table 3.** The full and minimally adequate linear regression models with coefficients (±SE) and significance levels (*≤5%, **≤1%, ***≤0.1%) for each retained variable, *R*^2^ = 0.11

Variables	Full model	Final model
Being French	−0.498 (±0.154)	−0.471 (±0.109)***
Previous experience (of insect food)	0.885 (±0.123)	0.937 (±0.107)***
Exercise (more than once weekly)	0.344 (±0.122)	0.268 (±0.108)*
Constant	0.691 (±0.340)	0.544 (±0.192)**
Frequent ‘Green’ shopper	0.208 (±0.340)	
Income	−3.250 × 10^–6^ (±1.640 × 10^–6^)	
High education level	−0.0464 (±0.177)	
Being British	0.0111 (±0.173)	
Gender	−0.190 (±0.118)	
Age	0.00131 (±0.00414)	

**Table 4.** Full and minimally adequate Heckman two-step models with coefficients (±SEs) and significance levels (*≤5%, **≤1%, ***≤0.1%) for each retained variable

WTP model	Full model	Final model
Age	0.0154	0.0147 (±0.00472) **
Frequent ‘Green’ shopper	0.217	0.2935 (±0.215)***
Mills’ ratio	3.029	2.935 (±0.215) ***
Gender	0.0516	
Being British	−0.238	
Being French	−0.111	
Exercise (more than once weekly)	0.210	
Income	1.130 × 10^–6^	
Previous experience (of insect food)	0.141	
Meat eater	−0.0137	
High education level	−0.227	
Market entry model		
Being French	−0.379	−0.444 (±0.099)***
Previous experience (of insect food)	0.675	0.693 (±0.094)***
Exercise (more than once weekly)	0.220	0.278 (±0.096)**
Meat eater	0.374	0.375 (±0.160)*
Income	−3.370 × 10^–6^	−3.080 × 10^–6^ (±1.250 × 10^–6^)*
Constant	−0.548	−0.653 (±0.179)*
Gender	−0.172	
Age	−0.0041	
Being British	0.110	
High education level	0.0347	
Frequent ‘Green’ shopper	0.126	

Only 11% of the variation in WTP was explained by the linear regression model and the value of the Mill’s ratio prompted a preference for the two-step Heckman method in characterizing variables underlying WTP. All the significant variables in the linear regression were also significant in the market entry model. ‘Age’ and ‘high frequency of green shopping’ were then identified as WTP explanatory variables once market selection bias was corrected. More than a third of respondents would consider buying ground/minced insect protein. The average WTP was £1.11 (±0.051) and the average fitted WTP was £1.18 (±0.017) for the linear regression. The average nonparametric WTP was £3.07 (±0.61) for market participants and the average fitted WTP was £3.60 (±0.0092) in the two-step Heckman model.

#### Choice Experiment

When offered a choice of two insect products and ‘neither’, >50% of respondents chose an insect alternative. Demographic characteristics were included and interactions with attributes were estimated (see Table 5). People exercising regularly or who had already eaten insect food before were more likely to choose an alternative where insects were visible. On the other hand, British people, younger people, meat eaters, regular green shoppers, postgraduate degree holders, and females were less likely to choose alternatives with visible insects. The willingness to accept compensation for eating insect food with visible insects over food with no visible insects was β_visibility_/β_price_ = 2.189/0.188 = £11.65.

**Table 5.** Significant coefficients in the conditional logit model for the choice experiment (*≤5%, **≤1%, ***≤0.1%), *R*^2^ = 0.263

Attributes	Coefficient (±SE)
Price	−0.188 (±0.0207)***
Visibility of insects	−2.189 (±0.329)***
Readiness to eat	0.156 (±0.0728)*
Interaction terms	
** **Visibility × age	0.0375 (±0.00476)***
** **Visibility × gender	−0.728 (±0.131)***
** **Visibility × British	−0.592 (±0.165)***
** **Visibility × meat eater	−0.864 (±0.252)***
** **Visibility × previous experience	0.687 (±0.138)***
** **Visibility × exercise more than once a week	0.363 (±0.137)**
** **Visibility × green shopping every time	−0.347 (±0.161)*
** **Visibility × postgraduate degree	−0.311 (±0.137)*

#### Potential Promotional Strategies

**Fig. 2.**
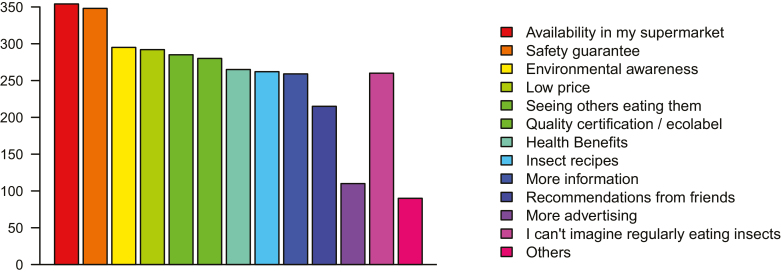
The number of survey respondents (from *N* = 1,020) citing the factors identified as making them more likely to eat insects regularly.

**Fig. 3.**
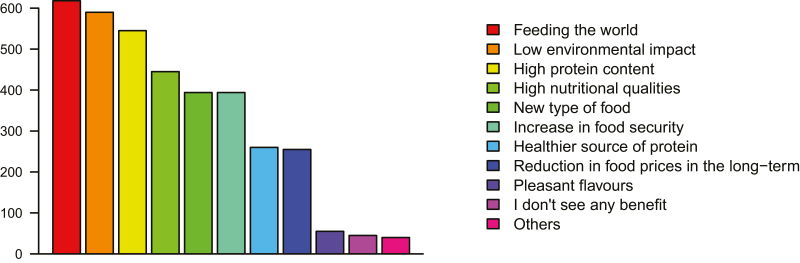
The number of survey respondents (from *N* = 1,020) citing each identified benefit of insect food.

In the hypothetical scenario presented, a greater proportion of people was willing to try insect food when offered it by a friend, 2/5 more than when it was given as a taster in a supermarket (χ^2^ = 8.1, df = 1, *N* = 922, *P* < 0.01). One-off ‘trying’ of an energy bar was contemplated by 55% of respondents, but only 8% could envisage buying them regularly. Three quarters of respondents predicted that chef or celebrity endorsement would make their friends more likely to eat insects. Overall, only one-fifth of respondents said that they could not imagine regularly eating insects and supermarket availability and safety appeared as the top two considerations when thinking about regular insect consumption, though a real mix of other factors was also relevant (Fig. 2). The top benefits of insect food perceived by survey respondents, contribution to ‘feeding the world’, and ‘low environmental impact’ express some degree of global concern rather than personal gain, coming second to both the protein content and nutritional qualities of the food (Fig. 3). Only 5% of the respondents saw no benefit to insect food though more than one-fourth of respondents felt at least some enthusiasm toward raising insects at home for personal consumption.

## Discussion

### Where Are the Opportunities? The Effects of Age, Affluence, Nationality, and Gender

Supporting the hypothesis that children may be more likely to accept insect food, the study in schools may indicate that there is a ‘sweet spot’ age for insect marketing. As the 6- to 11-yr olds were more likely to show interest in the insect products, this is compatible with the idea that the consumer behavior of children forms as early as infancy to 12 yr of age (Valkenburg and Cantor 2001). Younger children may have responded positively to insect products as they seemed to evaluate each picture based on the type of food it resembled (‘Because of the flavour of orange things’, boy aged 6–7 yr) rather than being focused on the insect content. This potentially key marketing window may be important for several reasons. First, it offers a viable alternative to many high-sugar snacks commonly marketed to school-age children and which contribute to rising obesity levels (Matthews 2008). Second, it suggests that there would be a growing market in the future as children become adults and gain purchasing power. Third, it unravels a particularly powerful route to adult interest: ‘pester power’. It has been established that children exert a significant influence on supermarket shopping (Wilson and Wood 2004), not restricted to products for their own consumption (Jensen 1995), as is the case for food. It may, thus, be possible to target two demographics with a single approach.

Unlike in Verbeke’s Belgian sample however (*N* = 368, 2015), younger adults did not appear more prone to adopting insect food. This disparity may be due to cultural variation or the abundance in our sample of young participants. Here, being older was associated with a slightly higher WTP which is consistent with consumers aged 35–64 yr spending more on food, and specifically meat products, relative to consumers under 35 yr (U.S. Bureau of Labor Statistics 2016) and that people over 65-yr old were under-represented in our sample. Although income was statistically significant in the market entry model, the coefficient has no economic significance and thus offers no support to the claim that insects are eaten by the ‘wealthy elites’ (Ramos-Elorduy 2009). The belief that higher income positively influences green food shopping may, thus, be a presumption; differences in income were also not relevant when examining determinants of green purchases for 547 Swiss consumers for example (Tanner and Kast 2003).

Unlike the children, where girls were less resistant than boys, adult women and men were similarly likely to be willing to pay for insect food. This may seem counter-intuitive as women are more likely to make health-conscious dietary changes (Fagerli and Wandel 1999) and insects are promoted as healthy products. Women, however, also feel more disgust to perceived ‘meatier’ attributes in some cases (Kubberød et al. 2002) and were here found to be less likely to tolerate visible insect parts; this may affect the appeal of insect food as a health food. The slight distinction seen between boys’ and girls’ attitudes is, in this study, possibly confounded with family background as most boy respondents came from School B which had different socioeconomic characteristics to School A.

Differences observed here between French and British consumers suggest that, within-Europe, culture may influence the attitude to insect food. French respondents placed less value on the benefits of insect food products whereas the British seemed more easily disgusted by the visibility of insects in products. Psychosocial influences are known to occur in the case of some green food products such as organic food (Baker et al. 2004) and cultural exposure is important in the evaluation of edible insects in particular (Tan et al. 2015). Consequently, some of the divergence with other single-country consumer surveys (Verbeke 2015) can be viewed in the light of localized psychosocial influences on food choice. The English place more importance on organic and convenience factors in their food decisions than the French, who prioritize pleasurable and social aspects of eating (Pettinger et al. 2004). Knowing this may help the industry adapt regional business models, although demographic factors may have a greater influence than the country of the consumers (Lennernäs et al. 1997). We should also consider that French participants may have had some shared unobservable characteristic as it is possible that difference in snowball recruitment pattern contributed to observed nationality traits.

#### Lifestyle Factors: Exercise, Vegetarianism, Shopping Habits

Frequent exercise was a clear shared characteristic among market participants. As health is the major driver of exercise, it is easy to see why healthy and protein-rich foods would appeal to this group (Prichard and Tiggemann 2008). Targeting people who exercise regularly could help boost insect sales as the use of dietary supplements, including protein shakes and bars, is prevalent in this market (Morrison et al. 2004). Case studies with other food products show that pleasant organoleptic experience, with positive taste, color, and smell evaluations may open possibilities of securing a regular customer base (Espejel et al. 2008). Indeed, appealing to the senses is crucial to triggering gastronomic interest in insect products (Deroy et al. 2015).

In general, meat-eaters were more likely to be willing to pay for insect food than vegetarians. As vegetarianism is most often adopted due to concerns about health and the ethical treatment of animals (Fox and Ward 2008), insect protein may still appeal to some who have chosen vegetarianism for these reasons. Vegetarians will be a minority of consumers, but some of them may well consider insects as a protein alternative.

Insect food is usually marketed as a sustainable product, but literature suggests that environmental arguments are limited in promoting ‘ecological’ eating (Tobler et al. 2011, Berger et al. 2018). Indeed, stated green habits in the parents’ questionnaires did not translate into an increased willingness to try insect food. Yet, green shoppers were more likely to enter the insect food market than others when green habits were framed in terms of green shopping frequency. In addition, environmental awareness was the third stated factor in making participants more likely to consume insects regularly which supports an effect for green shopping. Nevertheless, there may be a value-action gap for insect products, as exists for some other sustainable food products (Vermeir and Verbeke 2006).

Survey and questionnaire for participants identified altruistic and global benefits to insect food, but consumer purchase motivations are usually self-centered, even in the case of green products (McEachern and McClean 2002). Economic theory predicts that, collectively, environmentally friendly goods are preferred, yet individuals may be expected to reveal different preferences when individual economic utility is at play (Uusitalo 1990). Even if a web format is used and linked to more accurate reporting (Kreuter et al. 2008), it is possible that social approval pushed some respondents to overstate the importance they put on environmental and ethical dimensions in this online survey. Consequently, caution should prevail when looking at the magnitude of the identified effect of green shopping habits. Stated consumer WTP for insect products may not fully translate into market action as the low market shares for ethical food suggest (Young et al. 2010). Marketing strategies outside the green sphere will have to be considered if insect food is to become ‘mainstream’.

### Marketing Considerations

#### Information

Both survey and questionnaire showed a broad awareness of insects as food and demonstrated that providing more information can improve the likelihood that people will want to try entomophagy. Though most are aware of the practice, thanks to media coverage of the topic, they know less about the nutritional benefits of insect food or the range of possible products. Providing relevant information worked in this study and the number of students willing to try insects rose at the end of the school visits. Label information affects food purchase intentions (Baixauli et al. 2008) and simply participating in a survey about entomophagy can improve the attitude of participants toward insect food (Lensvelt and Steenbekkers 2014). Having previous experience, and thus having direct sensory information, was also a noteworthy variable in the market models. Different types of information are, thus, relevant and potentially synergistic, which gives support for greater public communication about the benefits of insect food and the limits of environmental resources.

Another aspect of information to be considered is safety as European consumers will need reassurance on product safety to gain consumer trust. In addition to this, the second-highest consumer concern identified here and one of the main market barriers seems to simply be the lack of availability in shops. Occasions to try insect foods are constrained by purchasing opportunity, which suggests that market growth may occur as products become more readily available to mainstream shoppers.

#### Peers and Other People

Although some Western consumers do not think the information provided by famous people on entomophagy is trustworthy (Lensvelt and Steenbekkers 2014), celebrities or high-profile environmental advocates may very well represent valuable supporters of the insect food movement. Survey respondents here predicted a positive effect of celebrity endorsement, and thus, advocacy by environmentally minded celebrities interested may offer a welcome push to the industry. Peer relationships confirm important social dynamics, thus rationalizing the practice of peer-to-peer marketing in the case of edible insects. Here, ‘seeing others eat insects’ was more influential than knowing about their nutritional benefits or health value. Moreover, greater willingness to try the food when offered by a friend compared with a supermarket employee was clear. Consumers may not think that recommendations from friends are very likely to make them buy insect food regularly, but talking with friends is a common way to gain product information (Bloch et al. 1986).

Young children were positively influenced by talking about the insect food pictures with their peers. Hence, peer influencing via digital marketing may be particularly successful when targeting a technophile young audience (Spero and Stone 2004). Though we found no positive peer influence between teenagers in this study, this may be because teenagers can be unwilling to admit it, as Lee (2014) found that the strongest predictor of the green shopping behavior of Hong Kong adolescents was peer influence

### Food Attributes

#### Presentation and Price

Insect products are currently mostly small snack items and there may be an assumption among manufacturers that snacks are an effective way to promote insect products. Here, we could not establish any preference for snacks over meals and insect dishes in children, who consume more snacks than adults (Jahns et al. 2001, Piernas and Popkin 2010). Snacks may be used for practical reasons to introduce people to insect food as the market develops, but there seems to be no reason on the consumer side why the industry should restrict itself to these items. Showing pictures of fully cooked dishes with insects elicited the same responses as when children looked at pictures of insect snacks. In both cases, presentation, anticipated texture, or expected taste were all important aspects on top of the overriding consideration of whether insects were visible. The substantial £11 penalty indicates that most consumers are not willing to trade-off price and visibility of insect parts when choosing these products. Visible insect parts are a strongly negative attribute, seen here in the children’s insect food picture ranking and in the survey of Dutch and Australian consumers by Lensvelt and Steenbekkers (2014). This is one reason why even some low-priced insect products may not sell well. As the market develops and more people are familiarized, products with whole or recognizable insects may yet become more viable as the positive interaction between visibility and previous experience does indicate that those who may have enjoyed insect food in the past will be less reluctant to buy products where insects are an obvious ingredient.

For now, the choice experiment indicates clearly that manufacturers may want to consider products where the nature of the food is not apparent. Insects used as an ingredient in the form of a powder that can be added to baking preparations, cooking sauces, etc., or sold in a prepared, processed form, such as insect burgers will find greater favor with the general consumer. This preference for familiar-looking food suggests that ready-meals with insects may find a market. In the United Kingdom, insect products could then conveniently use to their advantage of the two prevalent trends in this market: health food and convenience food (Shiu et al. 2004).

As Lensvelt and Steenbekkers also suggested (2014), price matters in insect food purchasing decisions. When choosing food, one major concern may drive the decision (Scheibehenne et al. 2007) and strong preferences for green products may only occur when trade-offs are not apparent (Olson 2013). One apparent trade-off, the visibility of insect parts, was of greater influence than price in the choice experiment highlighting that different factors will influence purchase decisions: the insect market is far from being one-dimensional.

The average WTP for 500 g of mealworm protein was less than half that of the conventional meat product. Nevertheless, the subset of participants with a positive WTP was willing to pay a comparable amount to the price of traditional minced meat. Consequently, different market sections could emerge based on price differentiations. Early adopters may be interested by medium/high-end items, whereas insects may only become an option for some if prices go down. As farming techniques develop, the price of insect food will fall and considerable market openings, from consumers who would be willing to pay for insect food at a low price only, may materialize.

### Industry Trends

This work counters the sometimes pessimistic view of the future of the insect food industry (Vanhonacker et al. 2013) and supports an encouraging vision outlined by others (Caparros Megido et al. 2014). This work extends the view of consumer opinions, supplementing the previous consumer base (Vanhonacker et al. 2013, Lensvelt and Steenbekkers 2014, Tan et al. 2015). The school investigation highlights the scope to create consumer favor for insect food and the online survey shows that the potential consumer base may be larger than previously thought. Most consumers see benefits in insect food and were willing to choose between insect food products in the hypothetical set-up. In many instances, children minded more what the insects were served with or what the dish was than the fact that the dish contained insects (‘I would eat it if I liked quiche’, boy in year 2). Finally, the portion interested by the idea of raising insects at home for personal consumption provides further support to say there are genuine market opportunities.

#### Constraints of This Work.

A limitation of our school investigation is the reliance on pictures rather than tasting of real products, due to both ethical and practical reasons. In addition, having schools with different socioeconomic backgrounds was useful in helping to extrapolate the results to the general population but may also confound some of the observed differences. Further, more extensive work would clarify this and could include rural populations too as specific food intakes vary between rural and urban children (Kirby et al. 1995).

Although the large sample size increased reliability, the population surveyed here was not representative of Western consumers as a whole. Income was similar to the median national annual earnings in the United Kingdom, but the sample was highly educated (85% degree-holders vs 36% in U.K. population; Office for National Statistics 2016). There also was a youthful skew in the age distribution; this young, educated population may have a more adventurous profile but also suggests that there will be a growing market in the future as increasingly familiarized children and young adults become more active on the consumer market.

#### Econometric Models

Even with great care taken in creating contingent valuation scenarios, hypothetical bias leads to individuals having a tendency to overstate their WTP (List 2001); the results obtained are, thus, based on intentions and not real market actions. This sample may be expected to have had more environmentally friendly intentions than a wider population sample due to the education and sample selection bias. Our use of convenience sampling strengthens the caution needed when extrapolating too widely from these results.

In addition, there was some potential endogeneity in the two-step Heckman model. Previous experience (entering the market at *t* − 1) correlated with entering the market now and had the largest coefficient value of all significant model variables. In the absence of data for instrumental variables which may correlate with previous experience, for example traveling abroad, great care should be taken when interpreting the coefficients of the model variables. Further work could quantify the effects of the identified variables in influencing purchasing behaviors in the context of diverse insect foods. Despite possible endogeneity, the significance of Mill’s ratio emphasizes that the two-step model was more appropriate in modeling WTP than the simple linear regression.

### Summary

Taken together, these lines of evidence paint a promising picture of the insect market in the West, with room for product development. Market analysis and WTP reveal that the population segment interested is substantial and has the potential to grow. Specifically, this work indicates that there are opportunities for insect food to develop further in terms of product types (ingredients, whole insects, ready-meals) and price categories (gourmet items, medium, or low-end products as farming costs decrease). Considerable changes in market dynamics can be expected, particularly in Europe as confidence grows. There will be no single ‘silver bullet’ marketing strategy: different marketing strategies will have to be used according to the geographical location and associated consumer characteristics.

General guidance in the West is to make insects less visible in the products, educate consumers, and make use of social dynamics to promote products. As access to products expands, we may find that the ‘disgust factor’ is less strong than initially expected. This may be particularly true if the identified interested child segment is targeted early and educated about the benefits of insect food.

### Future Prospects

Consumer research is a key element in helping the market for insect food products grow and this field will need to expand. This study indicates that products could be better matched to specific markets; their range could be increased, and price is an essential product attribute. One avenue to explore further are the economic opportunities for insect food purchasing behavior; modeling projections of future meat prices and insect prices could indicate a price point at which insects could truly become ‘mainstream’. Investigating the processes underlying one-off to regular consumption patterns and how to drive sales using a taste approach also merit attention if insects are ever to be part of Western weekly diets.

Worldwide food systems, and in particular Western ones, are under increasing pressure to evolve and a holistic approach involving ‘people, business and government’ (Green et al. 2006) is recommended to address the sustainable consumption challenge, of which insects are a part. Collective and collaborative action is needed to make our food systems more sustainable (Grinnell-Wright et al. 2013) and cooperation between actors is more likely to happen if governments support the insect market. Direct engagement could occur, thanks to the health and environmental advantages of insects, including their potential ‘circular system’ contribution to reducing food-waste at domestic and commercial scales in urban areas (Livin Farms 2018). Indirect support includes developing legislation to include insect farming standards and best practice guidelines. Successful cases of industry–academia–government networks, for example, in the Netherlands where products are on supermarket shelves, will mentor the process of establishing influential collaborative networks.

## Disclaimer

This study was carried out in accordance with the recommendations of the Imperial College London approvals process for non-medical studies and was approved at the Departmental level by Chair’s review.

## Supplementary Material

saz015_suppl_Supplementary-Materials
