# Supplementary material for: Insect Food Products in the Western World: Assessing the Potential of a New ‘Green’ Market
Source: Ann Entomol Soc Am. 2019 Sep 11;112(6):518–28. doi: 10.1093/aesa/saz015 (PMC6847481; doi:10.1093/aesa/saz015)
Supplement: saz015_suppl_Supplementary-Materials [file saz015_suppl_supplementary-materials.pdf]

Title: Insect food products in the Western world: Assessing the potential of a new 'green' market

Authors: Collins, C.M.*, Vaskou, P., Kountouris, Y.

Centre for Environmental Policy, Imperial College London

Supplementary Materials

School study, insect food pictures

School study, insect food pictures

All pictures were presented individually on one full page to the children.

Category 'snacks with visible insects'

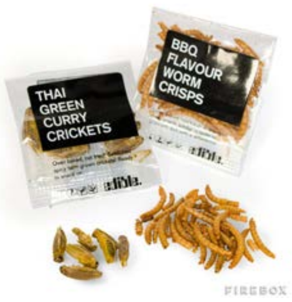

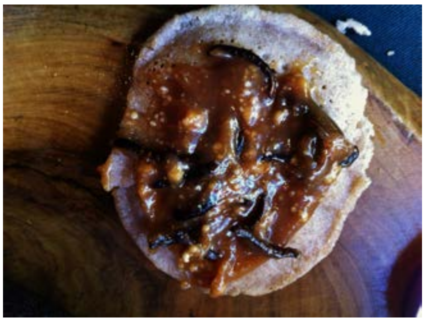

Insect snacks

Insect spread

Caramel-dipped locusts

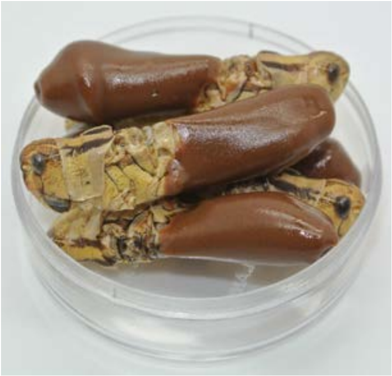

Category ‘snacks with no visible insect’

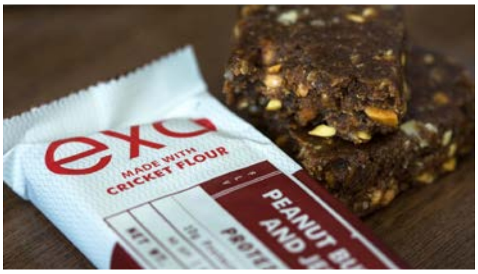

Insect bar

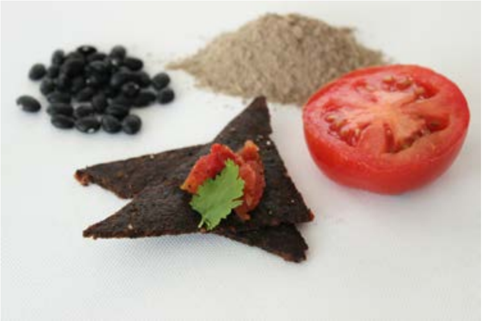

Cricket crisps with some of the ingredients

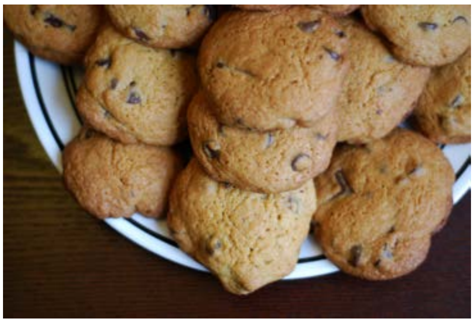

Cookies with cricket powder

Category 'dishes with visible insects'

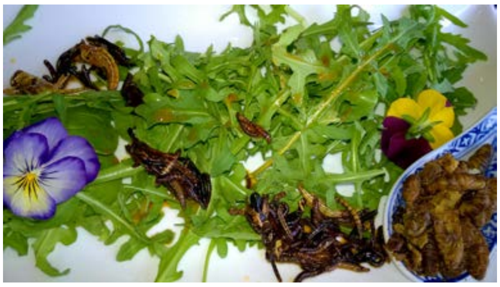

Bug salad

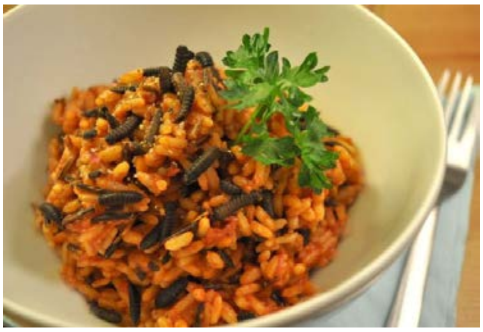

Fried rice with larvae

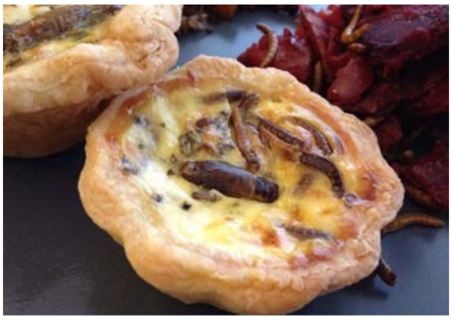

Insect quiche

Category ‘dishes with no visible insect’

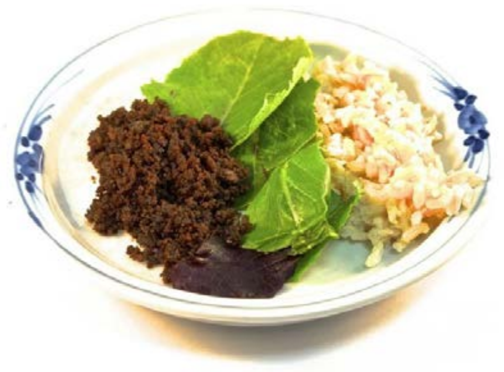

Mealworm protein with rice

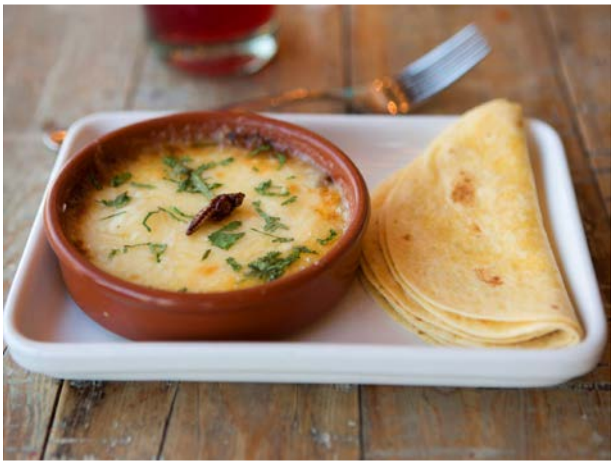

Grasshopper moussaka

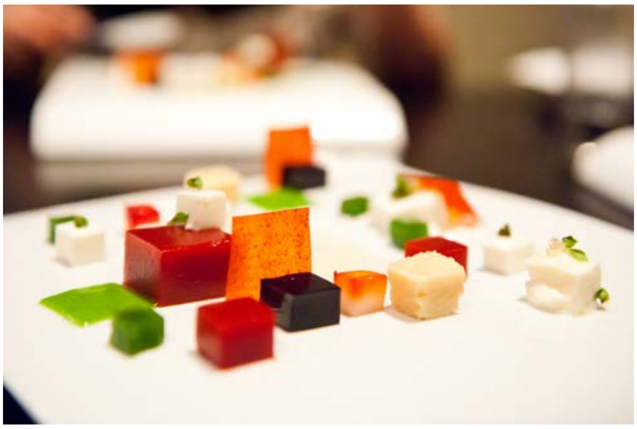

Insect cubes

7.2 School questionnaire children

Questionnaire - Student Research Project

Thank you for your help with my research project. All answers are confidential.

1) ID Number

**2) Are you**<br>□ Female<br>□ Male	**3) How old are you?**	**4) What is your nationality?**

**5) Do you eat meat?**	□ Yes □ No
**6) Have you ever eaten food made from insects?**	□ Yes □ No

The world population is increasing. We may not have enough meat for everyone in the future.

- Insects are high in protein, calcium and vitamins.
- Insects have a low impact on nature. They are healthier than red meat.
- They can be used in salads, stir-fries, curries, etc.

**7) Did your friends influence your opinion on insects in the small group?** (please tick)	□-5 □-4 □-3 □-2 □-1 □0 □+1 □+2 □+3 □+4 □+5<br>-5 = yes, in a negative way 0 = no +5 = yes, in a positive way
**8) Would you like to try insect food?**	□-5 □-4 □-3 □-2 □-1 □0 □+1 □+2 □+3 □+4 □+5<br>-5 = not at all 0 = unsure +5 = yes, a lot
**9) Can you imagine eating insects regularly?**	□-5 □-4 □-3 □-2 □-1 □0 □+1 □+2 □+3 □+4 □+5<br>-5 = not at all 0 = unsure +5 = yes, easily
**10) Do you like trying new things?**	□-5 □-4 □-3 □-2 □-1 □0 □+1 □+2 □+3 □+4 □+5<br>-5 = not at all 0 = neutral +5 = yes, a lot
**11) What would you prefer eating?**	□ an insect snack that your parents bought<br>□ a meal with insects cooked by your parents

7.3 School questionnaire parents

Parent Questionnaire - Student Research Project

(feel free to email pauline.vaskou11@imperial.ac.uk if you have any question)

Your child's class has been visited by a university student to talk about the future of food, as part of an MSc research project. We also talked about the scientific method and it would really help the class discussion if you answered this short survey for the next session. Please tick the appropriate answers.

All answers are confidential. Thank you for your help with my research project.

1) ID Number

**2) Are you**<br>□ Female<br>□ Male	**3) How old are you?**	**4) What is your nationality?**

**5) Do you eat meat?**	□ Yes □ No
**6) Have you ever eaten insects?**	□ Yes □ No
**7) If no, did you know that insects could be eaten for food?**	□ Yes □ No
**8) Do you have a particular interest in the environment or nature?**	□ Yes □ No

Examples of insect food can be found on the other side of this page.

The world population is increasing. We may not have enough meat for everyone in the future.

- Insects are high in protein, calcium and vitamins.
- Insects have a low impact on nature. They are healthier than red meat.
- They can be used in salads, stir-fries, curries, etc.

**9) Would you like to try insect food?** (please tick)	□-5 □-4 □-3 □-2 □-1 □0 □+1 □+2 □+3 □+4 □+5<br>-5 = not at all 0 = unsure +5 = yes, a lot
**10) Can you imagine eating insects regularly?**	□-5 □-4 □-3 □-2 □-1 □0 □+1 □+2 □+3 □+4 □+5<br>-5 = not at all 0 = unsure +5 = yes, easily
**11) Do you like trying new things?**	□-5 □-4 □-3 □-2 □-1 □0 □+1 □+2 □+3 □+4 □+5<br>-5 = not at all 0 = neutral +5 = yes, a lot
**12) Do you think insects can help feed the growing world population?**	□-5 □-4 □-3 □-2 □-1 □0 □+1 □+2 □+3 □+4 □+5<br>-5 = not at all 0 = unsure +5 = yes, a lot
**13) Do you care about how 'green' the food you buy is?**	□-5 □-4 □-3 □-2 □-1 □0 □+1 □+2 □+3 □+4 □+5<br>-5 = not at all 0 = unsure +5 = yes, a lot

7.4 Online survey, English version

English ▼

Introduction

Thank you for taking part in my student research project!

You will be given the opportunity to enter a £25 cash prize draw at the end of the 10mins survey.

Answers are all anonymous so please be honest.

Please click >> to start.

Demographics

The following questions are anonymous and used for statistical purposes only.

What is your gender?

- ◯ Male
- ◯ Female
- ◯ Prefer not to say

What is your nationality?

How old are you?

What is the highest level of education you have completed?

- ◯ Year 11/GCSE or equivalent
- ◯ 6th Form/College
- ◯ Diploma/undergraduate degree
- ◯

- Postgraduate degree
- Doctoral degree
- Prefer not to say

What is your annual household income before tax?

- less than £9 999
- £10 000 to £19 999
- £20 000 to £29 999
- £30 000 to £39 999
- £40 000 to £49 999
- £50 000 to £59 999
- £60 000 or above
- Prefer not to say

Habits

Do you eat meat?

- Yes
- No (state why in the box)

How often do you buy organic or eco-friendly food products?

Less than once a month	Once a month	A few times a month	Once a week	Every time I shop
○	○	○	○	○

How often do you exercise?

Less than once a month	Once a month	A few times a month	Once a week	More than once a week
○	○	○	○	○

Previous Experience

Are you aware that insects, such as crickets or ants, can be cooked for food?

- Yes
- No

Have you ever eaten insects before?

Yes	No
○	○

What did the food taste like?

	very poor					average					excellent
	-5	-4	-3	-2	-1	0	1	2	3	4	5
Slide the button											

Market Setting

Imagine you are doing your weekly shopping in your usual supermarket.

You are offered some cricket crisps for free.

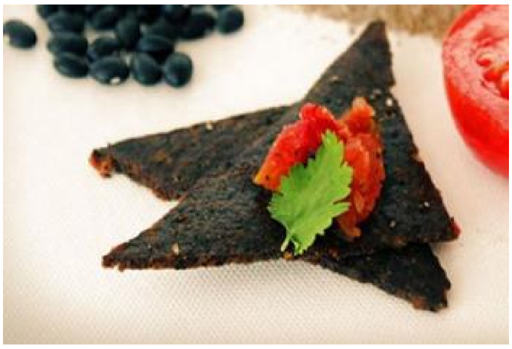

Would you try one?

Yes	No	Unsure
○	○	○

These page timer metrics will not be displayed to the recipient.
First Click: *0 seconds*
Last Click: *0 seconds*
Page Submit: *0 seconds*

Click Count: *0 clicks*

Imagine your best friend invites you for a barbecue.

Your best friend has bought some insect burgers from the supermarket.
He/she tells you "They're safe to eat and they taste really nice!", while showing you the packaging.

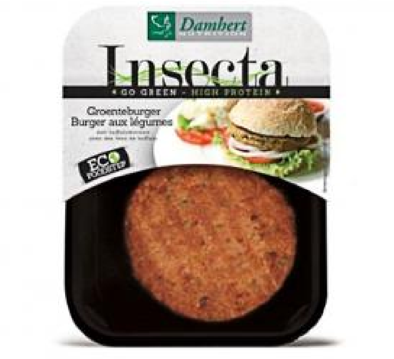

He/she then offers you a cooked insect burger. Would you try one?

Yes	No	Unsure
○	○	○

These page timer metrics will not be displayed to the recipient.

First Click: *0 seconds*

Last Click: *0 seconds*

Page Submit: *0 seconds*

Click Count: *0 clicks*

The world population is growing. Scientists expect we won't be able to provide enough protein if meat is produced and consumed as it is now in developed countries.

Insects such as crickets, grasshoppers, mealworms or ants can be cooked for food.

- They contain a lot of vitamin B2, vitamin B3, iron, omega-3s and calcium.
- Their protein content is higher than traditional meat sources.
- They are healthier than red meat.
- They are the most environmentally-friendly source of protein, with a low-carbon production.

Mealworm mince can be used in the same way as common meat mince.
Would you consider buying a 500g pack of mealworm mince?

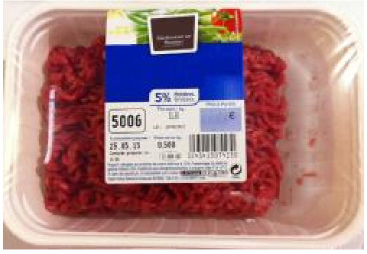

Yes

No

Considering a 500g pack of beef mince costs around £3.50, how much would you be willing to pay in pounds for the same amount of mealworm mince?

Keep in mind that x pounds spent on mealworm mince could not be spent on other things such as groceries or household expenses.

	1	2	3	4	5	6	7	8	9
Slide the button (£)									

Why would you not consider buying mealworm mince?

- I refuse to eat insects.
- I find the idea repulsive.
- I don't think it would be healthy.
- I don't think it would taste nice.
- I don't think it would help the environment.
- Other(s)
- It wouldn't work with my diet.

Choice Prices

Please select which product you would prefer buying in each case.

£3.50 grasshopper mince	£3.20 ready-to-cook crickets	
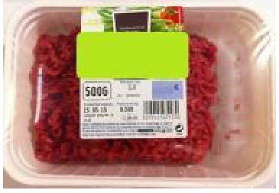	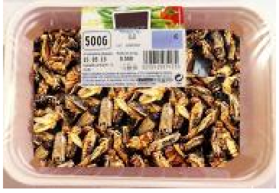	I would not buy either.

£5.00 mealworm-protein and rice	£4.80 fried rice with larvae	
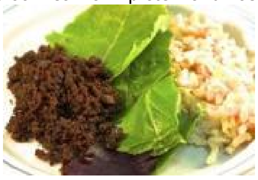	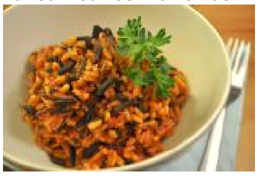	I would not buy either.

£4.90 insect quiche	£5.00 insect burger	
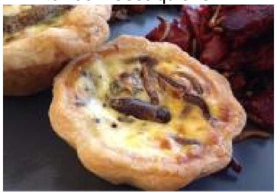	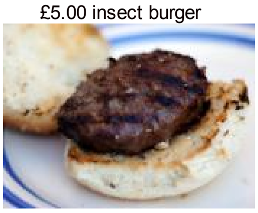	I would not buy either.

£1.00 caramel dipped locust snack	£1.40 cricket crisps	
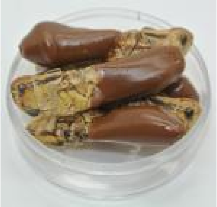	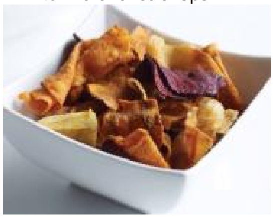	I would not buy either.

Marketing

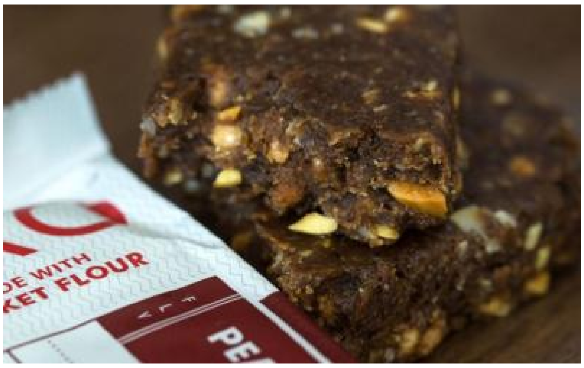

A new range of tasty energy bars made with insects has been launched.

They are easy to grab on the go and packed with energy to keep you going whenever needed!

Would you buy them?

- Yes, as a one off
- Yes, regularly
- I don't know
- No

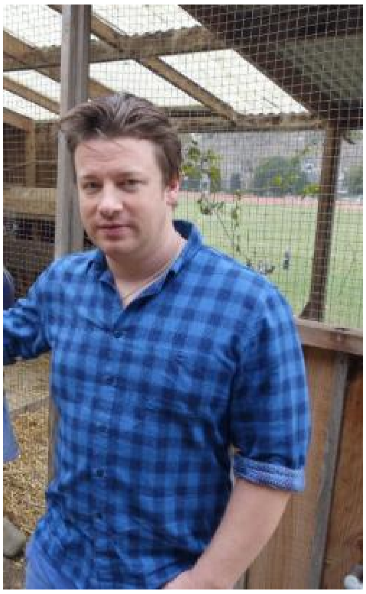

Imagine you're reading the news with your coffee.
You come across an article where Jamie Oliver writes about the benefits of cooking with insects.
It is called 'How to cook the new healthy superfood'.

In your opinion, would reading the article make your friends more likely to eat insects?

Not at all	A little bit	Quite a bit	A lot
○	○	○	○

What do you feel are the main benefits of insect food?
(select all that apply)

- [ ] new type of food
- [ ] high nutritional qualities
- [ ] high protein content
- [ ] low environmental impact
- [ ] pleasant flavour(s)
- [ ] healthier source of protein
- [ ] increase in food security
- [ ] helps to feed the world
- [ ] reduction in food prices in the long-run
- [ ] other(s)
- [ ] I don't see any benefit.

Which factors would make you more likely to **regularly** eat insects?
(select all that apply)

- [ ] seeing others eat them
- [ ] health benefits
- [ ] availability in my supermarket
- [ ] quality certification/ecolabel
- [ ] recommendations from friends
- [ ] more information
- [ ] environmental awareness
- [ ] insect recipes
- [ ] more advertisement
- [ ] low price
- [ ] safety guarantee
- [ ] other(s)
- [ ] I can't imagine regularly eating insects.

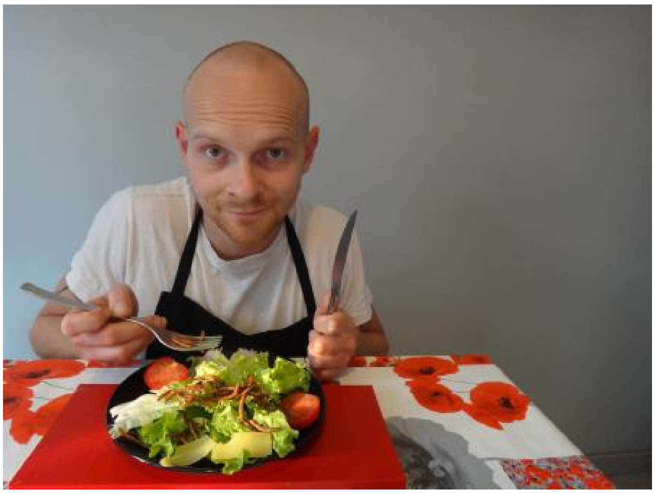

Around a quarter of road freight is due to food.
Insects could easily be raised at home for personal consumption, using a small container.
How enthusiastic would you be about being able to raise your own?

	not at all					neutral					very
	-5	-4	-3	-2	-1	0	1	2	3	4	5
Slide the button											

End

Do you live in Europe, the USA or Canada?

- Yes
- No
- Prefer not to say

Use the box below for any additional comment you may have.
Leave your email address if you wish to be entered in the £25 prize draw for taking part.
Click on >> to finish the survey.
